# Impacts of temperature and pH on the distribution of archaeal lipids in Yunnan hot springs, China

**DOI:** 10.3389/fmicb.2013.00312

**Published:** 2013-10-30

**Authors:** Weiyan Wu, Chuanlun L. Zhang, Huanye Wang, Liu He, Wenjun Li, Hailiang Dong

**Affiliations:** ^1^State Key Laboratory of Marine Geology, Tongji UniversityShanghai, China; ^2^Department of Marine Sciences, the University of GeorgiaAthens, GA, USA; ^3^State Key Laboratory of Loess and Quaternary Geology, Institute of Earth Environment, Chinese Academy of SciencesXi’an, China; ^4^Graduate School of Chinese Academy of SciencesBeijing, China; ^5^Key Laboratory of Microbial Diversity in Southwest China, Ministry of Education, Yunnan Institute of Microbiology, Yunnan UniversityKunming, China; ^6^State Laboratory of Geobiology and Environmental Geology, China University of GeosciencesBeijing, China; ^7^Department of Geology and Environmental Earth Science, Miami UniversityOxford, OH, USA

**Keywords:** *Archaea*, GDGTs, organic proxies, temperature, pH, hot springs, Yunnan

## Abstract

In culture experiments and many low temperature environments, the distribution of isoprenoid glycerol dialkyl glycerol tetraethers (GDGTs) commonly shows a strong correlation with temperature; however, this is often not the case in hot springs. We studied 26 hot springs in Yunnan, China, in order to determine whether temperature or other factors control the distribution of GDGTs in these environments. The hot springs ranged in temperature from 39.0 to 94.0°C, and in pH from 2.35 to 9.11. Water chemistry including nitrogen-, sulfur-, and iron species was also determined. Lipids from the samples were analyzed using liquid chromatography–mass spectrometry (LC–MS). Distributions of GDGTs in these hot springs were examined using cluster analysis, which resulted in two major groups. Group 1 was characterized by the lack of dominance of any individual GDGTs, while Group 2 was defined by the dominance of GDGT-0 or thaumarchaeol. Temperature was the main control on GDGT distribution in Group 1, whereas pH played an important role in the distribution of GDGTs in Group 2. However, no correlations were found between the distribution of GDGTs and any of the nitrogen-, sulfur-, or iron species. Results of this study indicate the dominance of temperature or pH control on archaeal lipid distribution, which can be better evaluated in the context of lipid classification.

## INTRODUCTION

Isoprenoid glycerol dialkyl glycerol tetraethers (GDGTs) as essential membrane lipids of *Archaea* widely occur in natural environments (Schouten et al., 2013 and references therein). They can contain up to eight cyclopentyl rings (Trincone et al., 1988; Schouten et al., 2000, 2007). GDGTs with fewer than six cyclopentyl rings (except for GDGT-4) are ubiquitous and often abundant in low temperature environments (see reviews by Pearson and Ingalls, 2013; Schouten et al., 2013). However, GDGTs with five to eight cyclopentyl moieties are mainly found in geothermal environments (Schouten et al., 2007; Boyd et al., 2013; Li et al., 2013). In addition to these GDGTs, a structurally unusual GDGT, thaumarchaeol that contains one cyclohexyl ring in addition to four cyclopentyl rings is also widespread (Pearson et al., 2004, 2008; Zhang et al., 2006; Schouten et al., 2007, 2013; Pitcher et al., 2009a; He et al., 2012; Boyd et al., 2013; Li et al., 2013).

Proxies based on GDGT distribution have been shown to correlate with physical, chemical, or biological parameters. Studies of thermophilic cultures showed that the number of rings (ring index, RI) increased with increasing temperature (Uda et al., 2001; Shimada et al., 2008; Boyd et al., 2011) and decreased (Shimada et al., 2008) or increased (Boyd et al., 2011) with decreasing pH. In normal marine sediments thaumarchaeol and its isomer are predominantly from deposition of planktonic *Thaumarchaeota*; whereas in gas hydrate- or cold seep-dominated environments, methanotrophic *Archaea* performing anaerobic oxidation of methane constitute the majority of the archaeal community, which synthesize mostly GDGTs-1, -2, and -3 but not thaumarchaeol and its isomer. Based on the relative abundances of GDGTs-1, -2, and -3 and thaumarchaeol and its isomer, a methane index (MI) was developed as an indicator for anaerobic oxidation of methane associated with destabilization of gas hydrates (Zhang et al., 2011). Furthermore, the ratio of thaumarchaeol (Thaum) to GDGT-0 (caldarchaeol) correlated with the relative abundance of *Thaumarchaeota* to *Euryarchaeota* in natural settings (Zhang et al., 2006; Turich et al., 2007; Yang et al., 2010), which is consistent with the notion that thaumarchaeol is the biomarker for *Thaumarchaeota* and GDGT-0 the major component of euryarchaeotal membrane lipids (Schouten et al., 2007).

In geothermal springs of Yellowstone, Great Basin, Kamchatka, and Tibet, multiple factors vary simultaneously, and GDGT distribution shows various correlations with environmental or biological parameters in different regions (Pearson et al., 2008; He et al., 2012; Boyd et al., 2013; Li et al., 2013; Paraiso et al., 2013). The goal of this study was to determine how GDGT distribution may be affected by multiple parameters such as temperature, pH, and nutrients in Yunnan hot springs. Cluster analysis was performed to show the patterns of GDGT distribution in these hot springs, which resulted in two major groups (Group 1 and Group 2). Regression analysis showed that these two groups had distinct responses to temperature and pH, suggesting that impacts of environmental variables on the distribution of GDGTs need to be evaluated in the context of lipid classification.

## MATERIALS AND METHODS

### FIELD SAMPLING

**FIGURE 1:**
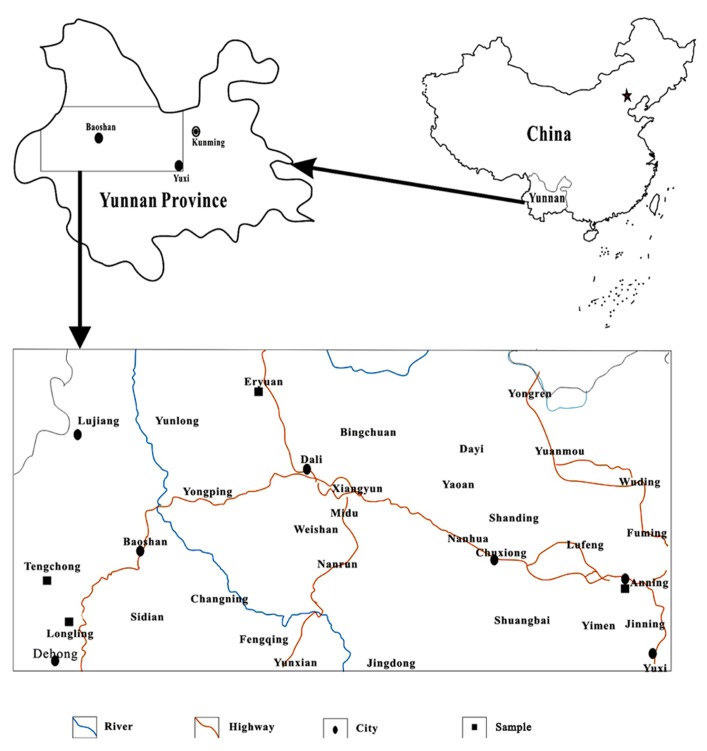
**A geographic map of the locations of hot springs in Yunnan, China**.

Twenty-six samples were collected from hot springs in Yunnan, China. Ten of them were from Rehai geothermal field in Tengchong (TC), nine from Longling (LL), six from Eryuan (EY), and one from Anning (AN; **Figure 1**). Temperature and pH were determined using a portable thermometer and pH meter, respectively. The concentrations of ammonium ${NH}_{4}^{+}$, nitrite ${NO}_{2}^{-}$, nitrate ${NO}_{3}^{-}$, sulfate ${SO}_{4}^{2-}$, and ferrous iron (Fe^2+^) were measured in the field by portable Hach kits according to the manufacturer’s instructions (Hach Company, Loveland, CO, USA).

Microbial mats and surface sediments were sampled by a hand scoop, which was pre-sterilized with 75% ethanol and dried before each new sample was taken. Samples were collected into sterile 50 ml Falcon tubes, immediately preserved in liquid nitrogen in the field, and stored at -80°C in the laboratory until further analysis.

### ARCHAEAL LIPID EXTRACTION

Samples were freeze-dried and ground to fine powder. Approximately 5 g of each powder sample was used to extract lipids according to a modified Bligh–Dyer technique (Bligh and Dyer, 1959; Zhang et al., 2012). A single phase solvent mixture (2:1:0.8, v/v/v) of MeOH, dichloromethane (DCM) and phosphate buffer (pH 7.4) was added to the sample in a centrifuge tube. The sample was sonicated (15 min) and then centrifuged (5 min, 2500 rpm). The extract was collected into a labeled glass tube. This procedure was repeated twice. DCM and phosphate buffer were added to the combined extract at 1:1:0.9 (v/v/v), achieving phase separation. The bottom DCM phase was collected into a 40 ml glass tube. The residue was rinsed with DCM (twice) and the DCM phase was also transferred to the glass tube. Finally, the total DCM fraction containing the target lipids was dried under N_2_ gas. The extract was separated into core lipids (F_1_) and polar fraction (F_2_) via pre-activated silica gel column chromatography according to Pitcher et al. (2009b), eluting with 10 column volumes of hexane/ethyl acetate (3:1 v/v) and 5 column volumes of MeOH, respectively. A known amount of C_46_ GDGT internal standard (IS; Huguet et al., 2006) was added to both F_1_ and F_2_. F_1_ and F_2_ were dried under N_2_ gas, re-dissolved in hexane/isopropanol (99:1 v/v) and filtered through a 0.45-μm PTFE syringe filter for subsequent high-performance liquid chromatography-mass spectrometry (HPLC–MS) analysis. F_2_ was used to determine whether core lipids would be eluted in the polar lipids (Schouten et al., 2010). If there were, the core GDGTs detected in F_1_ and F_2_ were combined as the final core GDGTs. Because the samples were left at room temperature for an extended period of time after freeze-drying, the fidelity of the polar lipids was questionable, thereby polar GDGTTs were not analyzed.

### ANALYSIS AND QUANTIFICATION OF CORE GDGTs

Analysis of core GDGTs (the word “core” is omitted from now on) was performed according to Wei et al. (2011) using an Agilent 6460 HPLC joined to a MS. Separation was achieved on Alltech Prevail Cyano column (150 × 2.1 mm, 3 μm) maintained at 30°C. A 5-μl volume of each sample was injected. GDGTs were eluted isocratically with 99% hexane and 1% isopropanol for 5 min, followed by a gradient to 1.8% isopropanol for 45 min with a flow rate of 0.2 ml/min. The column was cleaned after each analysis by back-flushing hexane-propanol (90:10, v/v) for 10 min. The relative abundances of GDGTs were quantified by integration of the peak areas from extracted single-ion chromatograms and comparison with the peak area of C_46_ IS. The abundance of GDGT-4 was obtained after accounting for the isotope effect by thaumarchaeol on the peak area of GDGT-4 (He et al., 2012).

### STATISTICAL ANALYSIS

Cluster analysis was performed using the R program according to Li et al. (2013). The relative abundance of GDGTs was used to construct Euclidean distance matrices describing the dissimilarity of the lipid distributions between samples, generating a hierarchical clustering tree (Turich et al., 2007; Pearson et al., 2008; Li et al., 2013). To measure the robustness of the clustering results, principal component analysis was also performed using CANOCO for Windows version 4.5. Regression analysis and bivariate correlation analysis were conducted using the SPSS software to evaluate the relationships of GDGTs with environmental parameters. A significant correlation was defined when *P*<0.05.

### CALCULATIONS OF RI AND MI

RI was calculated following the equation modified from Schouten et al. (2007). MI was calculated by the equation defined by Zhang et al. (2011).

$$R\,I=\frac{\begin{array}{c} G\,D\,G\,T-1+2*G\,D\,G\,T-2+3*G\,D\,G\,T-3+4*G\,D\,G\,T\\ -4+5*G\,D\,G\,T-5+6*G\,D\,G\,T-6 \end{array}}{100},$$

$$M\,I=\frac{G\,D\,G\,T-1+G\,D\,G\,T-2+G\,D\,G\,T-3}{G\,D\,G\,T-1+G\,D\,G\,T-2+G\,D\,G\,T-3+T\,h\,a\,u\,m+T\,h\,a\,u\,m\,.\,i\,s\,o\,m\,e\,r}\,.$$

## RESULTS

### WATER CHEMISTRY

Hot springs in Yunnan, China, exhibited diverse physical and geochemical conditions (**Table 1**). For example, temperature ranged from 39.0 to 94.0°C and pH from 2.35 to 9.11. The concentration of sulfate varied from 5.0 to 500.0 mg/L and was below 200.0 mg/L in most springs. The concentrations of ammonium, nitrite and nitrate were below 0.34 mg/L, 0.17 mg/L, and 0.69 mg/L, respectively. The concentration of ferrous iron was no more than 1.00 mg/L with the exception of TC-2 (17.00 mg/L). The concentration of sulfide was no more than 4.80 mg/L. Overall, these hot springs were characterized as low inorganic energy sources (Zhang et al., 2008).

**Table 1:** Location and water chemistry of Yunnan hot springs.

Sample^a^	GPS		pH	*T*(°C)	SO_4_^2-^ (mg/L)	NO_2_^-^ (mg/L)	NO_3_^-^ (mg/L)	NH_4_^+^ (mg/L)	Fe^2+^ (mg/L)	S^2-^ (mg/L)
TC-1^b^	N 24°57’12.7”	E 98°26’17.4”	7.64	84.0	300.0	0.05	0.51	0.02	BDL	0.31
TC-2^b^	N 24°57’12.7”	E 98°26’17.4”	2.35	87.0	500.0	0.03	0.06	1.18	17.00	0.21
TC-3^b^	N 24°57’12.8”	E 98°26’17.5”	9.00	94.0	6.5	0.04	0.56	0.01	BDL	4.80
TC-4	N 24°57’12.9”	E 98°26’17.6”	9.00	94.0	6.5	0.04	0.56	0.01	BDL	4.80
TC-5^b^	N 24°57’12.10”	E 98°26’17.7”	3.50	89.5	70.0	0.03	0.19	0.34	1.00	0.04
TC-6	N 24°57’12.11”	E 98°26’17.8”	8.80	89.0	8.0	0.04	0.69	BDL	BDL	3.40
TC-7	N 24°56’59.6”	E 98°26’15.7”	6.78	78.0	40.0	0.14	0.41	BDL	0.06	0.10
TC-8^b^	N 24°56’59.6”	E 98°26’15.7”	6.69	64.8	33.0	0.16	0.47	BDL	0.04	BDL
TC-9	N 24°56’59.7”	E 98°26’15.8”	7.62	52.2	BDL	BDL	BDL	BDL	BDL	BDL
TC-10	N 24°56’59.8”	E 98°26’15.9”	4.50	72.0	12.0	0.05	0.47	BDL	0.02	BDL
LL-1	N 24°39’37.2”	E 98°43’11.9”	6.78	50.7	15.0	0.01	0.03	BDL	0.01	BDL
LL-2^b^	N 24°39’37.3”	E 98°43’11.10”	7.07	44.5	29.0	BDL	0.03	BDL	0.02	BDL
LL-3^b^	N 24°39’23.3”	E 98°40’03.4”	6.30	60.0	40.0	0.01	BDL	BDL	BDL	BDL
LL-4	N 24°39’23.4”	E 98°40’03.5”	6.30	60.0	40.0	0.01	BDL	BDL	BDL	BDL
LL-5	N 24°39’23.5”	E 98°40’03.6”	8.22	86.0	46.0	BDL	0.03	0.05	BDL	BDL
LL-6	N 24°39’23.6”	E 98°40’03.7”	9.11	82.0	42.0	0.01	0.02	BDL	BDL	BDL
LL-7^b^	N 24°39’23.7”	E 98°40’03.8”	6.67	55.0	47.0	0.01	BDL	0.02	BDL	BDL
LL-8	N 24°39’23.8”	E 98°40’03.9”	6.79	39.0	32.0	0.01	BDL	BDL	0.05	BDL
LL-9	N 24°39’23.8”	E 98°40’03.9”	8.01	50.0	100.0	0.07	BDL	BDL	0.03	BDL
EY-1	N 26°04’37.7”	E 100°01’56.9”	7.12	48.0	BDL	BDL	BDL	BDL	BDL	BDL
EY-2	N 26°04’37.8”	E 100°01’56.10”	7.43	50.2	BDL	BDL	BDL	BDL	BDL	BDL
EY-3^b^	N 26°15’01.2”	E 99°59’22.2”	7.92	70.0	30.0	0.06	0.29	0.14	0.03	BDL
EY-4	N 26°15’01.2”	E 99°59’22.2”	6.67	70.0	160.0	0.11	0.33	0.11	0.02	BDL
EY-5^b^	N 26°14’57.1”	E 99°59’31.0”	6.86	62.0	140.0	0.11	0.36	0.05	0.03	BDL
EY-6^b^	N 26°14’58.3”	E 99°59’32.6”	7.25	80.0	120.0	0.05	0.11	0.21	0.01	BDL
AN-1	N 24°57’36.0”	E 102°27’3.6”	7.07	43.5	5.0	0.17	0.26	0.06	BDL	BDL

a TC-1, Dagunguo; TC-2, Diretiyanqu; TC-3, Yanjingquan; TC-4, Yanjingquan; TC-5, Zhenzhuquan; TC-6, Gumingquan; TC-7, Dawumingquan; TC-8, Wumingxiaoxishangyou; TC-9, Wumingxiaoxizhongyou; TC-10, Hamazui; LL-1, Dahebianzhongyou; LL-2, Dahebianzaotang; LL-3, Banglazhangshangxiao #1; LL-4, Banglazhangshangxiao #1; LL-5, Banglazhangshangxiao #3; LL-6, Banglazhangshangxiao #5; LL-7, Banglazhangshangxiao #6; LL-8, Banglazhangshangxiao #8; LL-9, Xiaoxiaqiangxia; EY-1; Xiashankouchitang; EY-2, Xiashankoudaotian; EY-3, Niujieyongpingzaotang; EY-4, Niujieyongpingshougongjing; EY-5, Shibeicunlaizitang #1; EY-6, Shibeicunlaizitang #2; AN-1, Tianxiadiyitang.

b Chemistry that has been reported in Jiang et al. (2010).

*BDL, below detection limit; *T*, temperature.*

### GDGT CONCENTRATIONS AND GDGT-BASED PROXIES

**FIGURE 2:**
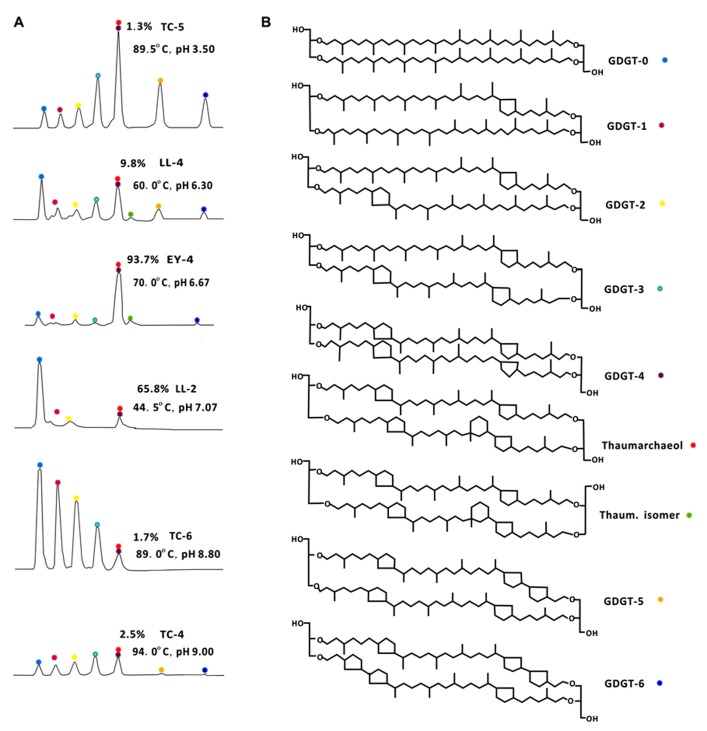
**HPLC–APCI–MS base peak chromatograms showing representative distributions of archaeal core lipids with temperature and pH values (A) and chemical structures of GDGTs from Yunnan hot springs (B).** TC-5, Zhenzhuquan; LL-4, Banglazhangshangxiao #1; EY-4, Niujieyongpingshougongjing; LL-2, Dahebianzaotang; TC-6, Gumingquan; TC-4, Yanjingquan. GDGTs from acidic hot springs with medium high temperature were dominated by GDGTs with more cyclopentyl rings, such as TC-5, LL-4, and EY-4, while those from neutral alkaline hot springs were dominated by GDGTs with fewer cyclopentyl rings, such as LL-2, TC-6, and TC-4. Thaum.isomer, Thaumarchaeol regioisomer. The relative abundance of thaumarchaeol to GDGT-4 for each sample was marked in each chromatograph.

GDGT-0 to GDGT-6, thaumarchaeol (Thaum) and its regioisomer (Thaum.isomer; **Figure 2**) were detected in Yunnan hot springs (**Table 2**). The concentration of the total GDGTs ranged from 1.43 to 993.83 ng/g; the most abundant GDGTs occurred in TC-5 and the least abundant in EY-3 (**Table 2**). The values of RI ranged from 0.14 to 3.99, those of Thaum/(Thaum + GDGT-0) from 0 to 0.89, and those of MI from 0.14 to 1.0 (**Table 3**).

**Table 2:** Relative abundances of GDGTs for core lipids from Yunnan hot springs.

Sample^a^	Total lipids (ng/g)	% GDGTs								
		GDGT-0	GDGT-1	GDGT-2	GDGT-3	GDGT-4	Thaum.	Thaum.isomer	GDGT-5	GDGT-6
TC-1^b^	331.5	36	20	15	16	13	0	0	1	0
TC-2^b^	501.7	2	2	*6*	13	51	0	0	15	12
TC-3^b^	92.5	5	6	11	23	52	1	0	1	0
TC-4	15.6	14	15	24	16	28	1	0	2	1
TC-5^b^	993.8	5	5	8	17	37	1	0	19	9
TC-6	706.1	34	29	29	2	7	0	0	0	0
TC-7	2.2	8	5	11	17	45	2	0	10	1
TC-8^b^	51.7	21	9	10	10	26	12	2	7	3
TC-9	11.9	42	4	4	5	17	24	0	3	0
TC-10	37.6	17	25	25	21	10	0	0	1	1
LL-1	1.8	28	7	8	4	2	51	0	0	0
LL-2^a^	28.4	75	2	1	1	7	14	0	0	0
LL-3^a^	193.1	33	9	11	15	24	4	1	2	1
LL-4	121.6	32	9	10	15	26	3	1	3	1
LL-5	418.1	11	9	14	24	40	1	0	1	0
LL-6	182.8	7	9	14	24	46	1	0	1	0
LL-7^b^	36.2	29	4	5	13	31	14	0	3	0
LL-8	28.8	14	5	9	12	25	34	0	0	0
LL-9	25.7	14	18	20	19	18	10	1	0	0
EY-1	57.4	75	2	3	2	0	17	1	0	0
EY-2	82.2	69	2	3	1	1	23	0	0	0
EY-3^b^	1.4	71	0	0	0	10	18	0	0	0
EY-4	22.5	10	3	6	3	4	65	7	0	1
EY-5^b^	43.3	49	3	3	2	3	35	4	0	0
EY-6^b^	68.4	73	2	*2*	1	8	15	0	0	0
AN-1	16.1	7	8	0	2	27	56	0	0	0

a TC-1, Dagunguo; TC-2, Diretiyanqu; TC-3, Yanjingquan; TC-4, Yanjingquan; TC-5, Zhenzhuquan; TC-6, Gumingquan; TC-7, Dawumingquan; TC-8, Wumingxiaoxishangyou; TC-9, Wumingxiaoxizhongyou; TC-10, Hamazui; LL-1, Dahebianzhongyou; LL-2, Dahebianzaotang; LL-3, Banglazhangshangxiao #1; LL-4, Banglazhangshangxiao #1; LL-5, Banglazhangshangxiao #3; LL-6, Banglazhangshangxiao #5; LL-7, Banglazhangshangxiao #6; LL-8, Banglazhangshangxiao #8; LL-9, Xiaoxiaqiangxia; EY-1, Xiashankouchitang; EY-2, Xiashankoudaotian; EY-3, Niujieyongpingzaotang; EY-4, Niujieyongpingshougongjing; EY-5, Shibeicunlaizitang #1; EY-6, Shibeicunlaizitang #2; AN-1, Tianxiadiyitang.

b Chemistry that has been reported in Jiang et al. (2010).

**Table 3:** Proxies based on GDGTs from Yunnan hot springs.

Sample^a^	Thaum/(Thaum + GDGT-0)	Methane index	Ring index
TC-1^b^	0.01	0.99	1.52
TC-2^b^	0.24	0.98	3.99
TC-3^b^	0.21	0.96	3.11
TC-4	0.05	0.99	2.35
TC-5^b^	0.09	0.98	3.67
TC-6	0.00	1.00	1.17
TC-7	0.20	0.95	3.19
TC-8^b^	0.37	0.67	2.14
TC-9	0.36	0.36	1.12
TC-10	0.03	0.99	1.89
LL-1	0.65	0.27	0.44
LL-2^b^	0.15	0.25	0.36
LL-3^b^	0.11	0.88	1.87
LL-4	0.08	0.89	1.97
LL-5	0.07	0.98	2.76
LL-6	0.08	0.99	2.94
LL-7^b^	0.33	0.61	1.92
LL-8	0.71	0.43	1.59
LL-9	0.40	0.84	1.88
EY-1	0.19	0.27	0.14
EY-2	0.25	0.19	0.16
EY-3^b^	0.20	0.00	0.41
EY-4	0.86	0.14	0.49
EY-5^b^	0.42	0.18	0.27
EY-6^b^	0.17	0.22	0.39
AN-1	0.89	0.15	1.23

a TC-1, Dagunguo; TC-2, Diretiyanqu; TC-3, Yanjingquan; TC-4, Yanjingquan; TC-5, Zhenzhuquan; TC-6, Gumingquan; TC-7, Dawumingquan; TC-8, Wumingxiaoxishangyou; TC-9, Wumingxiaoxizhongyou; TC-10, Hamazui; LL-1, Dahebianzhongyou, LL-2; Dahebianzaotang; LL-3, Banglazhangshangxiao #1; LL-4, Banglazhangshangxiao #1; LL-5, Banglazhangshangxiao #3; LL-6, Banglazhangshangxiao #5; LL-7, Banglazhangshangxiao #6; LL-8, Banglazhangshangxiao #8; LL-9, Xiaoxiaqiangxia, EY-1; Xiashankouchitang; EY-2, Xiashankoudaotian; EY-3, Niujieyongpingzaotang; EY-4, Niujieyongpingshougongjing; EY-5, Shibeicunlaizitang #1; EY-6, Shibeicunlaizitang #2; AN-1, Tianxiadiyitang.

b Chemistry that has been reported in Jiang et al. (2010).

### CLUSTER ANALYSIS

**FIGURE 3:**
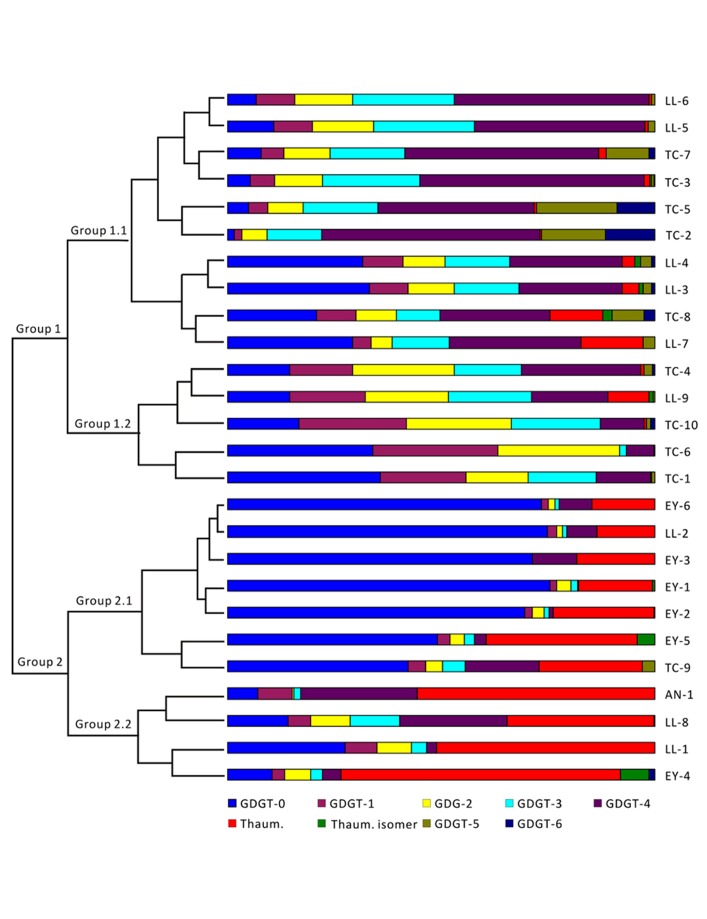
**Cluster analysis of archaeal lipids in hot spring sediments by R clustering method.** Sample names are shown on the right of the figure. GDGTs are color coded and shown at the bottom of the figure. TC-1, Dagunguo; TC-2, Diretiyanqu; TC-3, Yanjingquan; TC-4, Yanjingquan; TC-5, Zhenzhuquan; TC-6, Gumingquan; TC-7, Dawumingquan; TC-8, Wumingxiaoxishangyou; TC-9, Wumingxiaoxizhongyou; TC-10, Hamazui; LL-1, Dahebianzhongyou; LL-2, Dahebianzaotang; LL-3, Banglazhangshangxiao #1; LL-4, Banglazhangshangxiao #1; LL-5, Banglazhangshangxiao #3; LL-6, Banglazhangshangxiao #5; LL-7, Banglazhangshangxiao #6; LL-8, Banglazhangshangxiao #8; LL-9, Xiaoxiaqiangxia, EY-1, Xiashankouchitang, EY-2, Xiashankoudaotian, EY-3, Niujieyongpingzaotang, EY-4, Niujieyongpingshougongjing, EY-5, Shibeicunlaizitang #1; EY-6, Shibeicunlaizitang #2; AN-1, Tianxiadiyitang.

**FIGURE A1:**
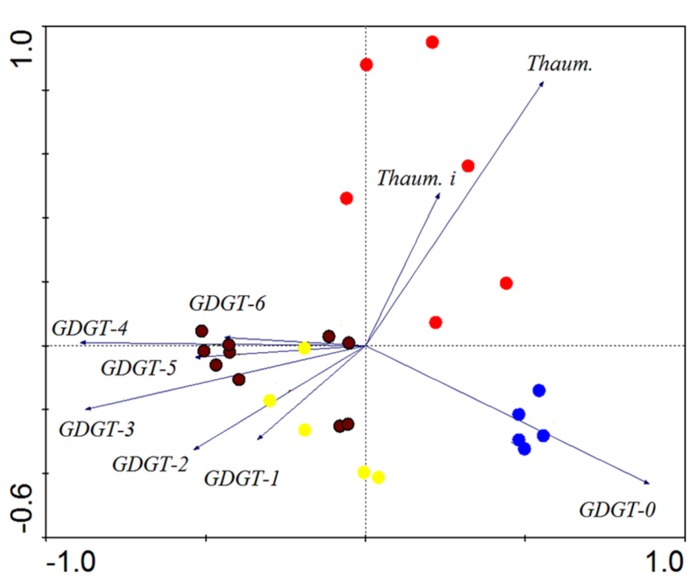
**Clustering results from Yunnan hot springs by principal component analysis.** Solid circles with brown, yellow, blue, and red color represent samples from Group 1.1, Group 1.2, Group 2.1, and Group 2.2 in **Figure 3**, respectively. Thaum, Thaumarchaeol, Thaum. i, Thaumarchaeol isomer.

Cluster analysis was conducted to examine the patterns of GDGT distribution. The results from R clustering method using Euclidean distance as the metric showed that all samples fell into two major groups (Group 1 and Group 2; **Figure 3**). The samples within Group 1 were primarily from Tengchong plus some samples from Longling. It was further divided into two subgroups (Group 1.1 and Group 1.2); Group 1.1 was distinguished from Group 1.2 by relatively more abundant GDGT-3 and GDGT-4. The samples within Group 2 were exclusively from Eryuan plus the remaining samples that were not found in Group 1. Group 2 was also divided into two subgroups (Group 2.1 and Group 2.2); Group 2.1 was defined by the dominance of GDGT-0 and Group 2.2 was characterized by the dominance of thaumarchaeol. The results were consistent with those from principle component analysis method using CANOCO for Windows version 4.5 (**Figure A1** in Appendix).

### CORRELATIONS OF INDIVIDUAL GDGTs AND ORGANIC PROXIES WITH ENVIRONMENTAL VARIABLES

**FIGURE 4:**
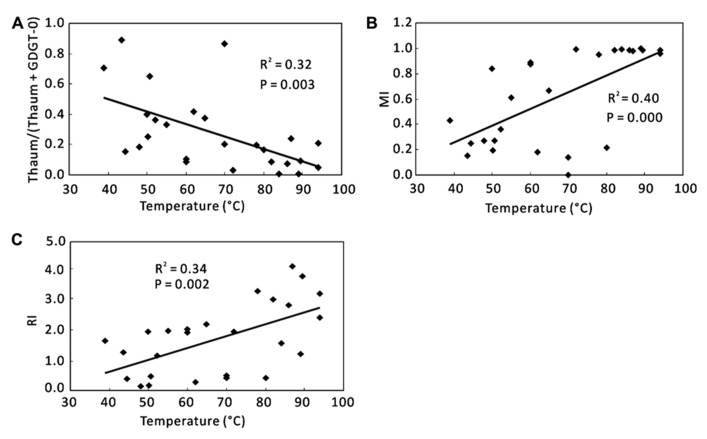
**Correlations between organic proxies and environmental parameters using total samples.** **(A)** Thaum/(Thaum + GDGT-0) and temperature; **(B)** MI and temperature; **(C)** RI and temperature. Thaum, Thaumarchaeol.

For total samples, correlations of individual GDGTs or organic proxies with environmental variables were examined. No significant correlations existed between individual GDGTs and any environmental variables (data not shown). However, some correlations were found between the organic proxies and temperature (**Figure 4**). Thaum/(Thaum + GDGT-0) showed a negative correlation with temperature (*R*^2^ = 0.32, *P* = 0.003; **Figure 4A**). MI (*R*^2^ = 0.40, *P* = 0.000; **Figure 4B**) and RI (*R*^2^ = 0.34, *P* = 0.002; **Figure 4C**) showed positive correlations with temperature. Significant correlations of these proxies with other factors such as pH and nutrients were not observed (data not shown).

**FIGURE 5:**
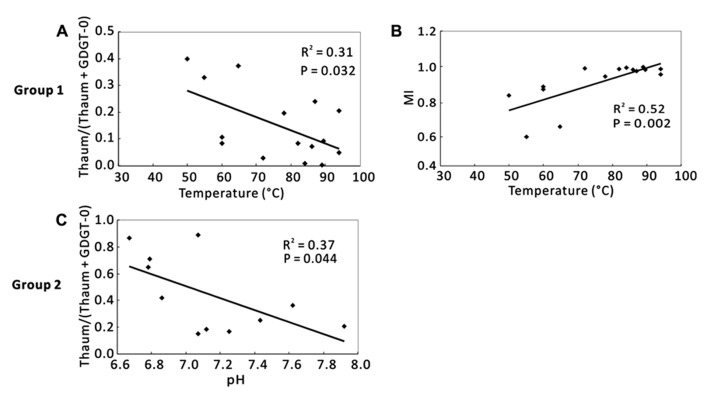
**Correlations between organic proxies and environmental parameters in two clustering groups according to Figure 3.** **(A)** Thaum/ (Thaum + GDGT-0) and temperature in Group 1; **(B)** MI and temperature in Group 1; **(C)** Thaum/(Thaum + GDGT-0) and pH in Group 2. Thaum, Thaumarchaeol.

When samples were divided into Group 1 and Group 2, temperature was the dominant factor for Group 1, and pH was the significant factor for Group 2 (**Figure 5**). In Group 1, Thaum/(Thaum + GDGT-0) negatively correlated with temperature (*R*^2^ = 0.31, *P* = 0.032; **Figure 5A**) and MI positively correlated with temperature (*R*^2^ = 0.52, *P* = 0.002; **Figure 5B**); the results were similar to the observation of the total samples (**Figures 4A,B**). In Group 2, Thaum/(Thaum + GDGT-0) negatively correlated with pH (*R*^2^ = 0.37, *P* = 0.044; **Figure 5C**). In either group, correlations between nutrients and the organic proxies were insignificant (data not shown).

## DISCUSSION

### ORIGIN AND DISTRIBUTION OF ARCHAEAL GDGTs FROM CLUSTER-GENERATED GROUPS

**FIGURE A2:**
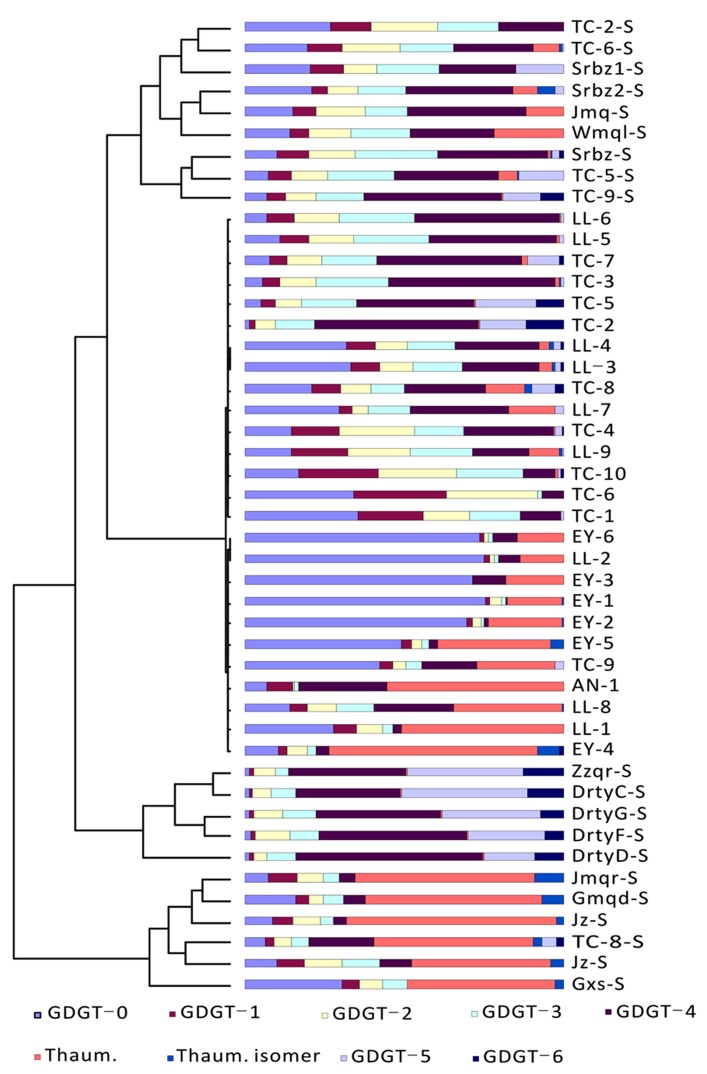
**Cluster analysis of archaeal lipids from Yunnan hot spring sediments in this study and surrounding soils (Xie et al., unpublished data).** Samples named with “S” are from soils surrounding hot springs. TC-1, Dagunguo; TC-2, Diretiyanqu; TC-3, Yanjingquan; TC-4, Yanjingquan; TC-5, Zhenzhuquan; TC-6, Gumingquan; TC-7, Dawumingquan; TC-8, Wumingxiaoxishangyou; TC-9, Wumingxiaoxizhongyou; TC-10, Hamazui; LL-1, Dahebianzhongyou, LL-2, Dahebianzaotang, LL-3, Banglazhangshangxiao #1; LL-4, Banglazhangshangxiao #1; LL-5, Banglazhangshangxiao #3; LL-6, Banglazhangshangxiao #5; LL-7, Banglazhangshangxiao #6; LL-8, Banglazhangshangxiao #8; LL-9, Xiaoxiaqiangxia; EY-1, Xiashankouchitang; EY-2, Xiashankoudaotian; EY-3, Niujieyongpingzaotang; EY-4, Niujieyongpingshougongjing; EY-5, Shibeicunlaizitang #1; EY-6, Shibeicunlaizitang #2; AN-1, Tianxiadiyitang; Srbz 1, Shuirebaozao #1; Srbz 2, Shuirebaozao #2; Jmq, Jiemeiquan, Wmql, Wumingquan left; Srbz, Shuirebaozao; Zzqr, Zhenzhuquan right; DrtyC, Diretiyan # C; DrtyD, Diretiyan #D; DrtyG, Diretiyan #G; DrtyF, Diretiyan #F; Jmqr, Jiemeiquan right; Gmqd, Gumingquan down; Jz, Jieming; Gxs, Gongxiaoshe.

Recent studies of hot springs in Tibet (He et al., 2012; Li et al., 2013), Yellowstone (Pearson et al., 2008; Boyd et al., 2013), and Great Basin (Pearson et al., 2008; Paraiso et al., 2013) revealed that GDGTs from theses hot springs were primarily produced autochthonously and not the result of soil contamination. Although soils surrounding Yunnan hot springs were not sampled in this study, they were collected in later field trips (Xie et al., unpublished), and showed an obvious difference in GDGT distribution from geothermal springs (**Figure A2** in Appendix), suggesting that GDGTs in the hot springs are dominated by those produced *in situ* rather than contamination from surrounding soils.

Studies based on pure cultures of thermophilic *Archaea* showed that the GDGT composition varies from species to species (Macalady et al., 2004; Schouten et al., 2007; de la Torre et al., 2008; Boyd et al., 2011). GDGTs with 0–4 cyclopentanyl rings are likely derived from thermophilic *Crenarchaeota* and *Euryarchaeota* (Schouten et al., 2007, 2013). GDGT-5 and GDGT-6 are produced predominantly by the orders *Sulfolobales* and *Thermoplasmatales*, which optimally grow at low pH (Schouten et al., 2007, 2013). Indeed, GDGT-5 and GDGT-6 occurred in acidic hot springs of Tengchong with abundant *Sulfolobales* and *Thermoplasmatales* (Hou et al., 2013), such as TC-2 and TC-5 in Group 1 (**Table 2**; **Figure 3**). Similar observations were made in Yellowstone (Pearson et al., 2008; Boyd et al., 2013), Kamchatka (Pearson et al., 2008), and Tibet (He et al., 2012; Li et al., 2013). Moreover, all retrieved archaeal species from TC-2 (Hou et al., 2013) and TC-5 (Song et al., 2010) were grouped into the *Sulfolobales* order. In summary, the distribution of archaeal membrane lipids corroborated 16S rRNA gene sequence data to show that *Archaea* in Group 1 hot springs were dominated by thermophilic *Crenarchaeota* and *Euryarchaeota*.

GDGT-0 has been identified in *Euryarchaeota*, *Crenarchaeota*, as well as *Thaumarchaeota* (Brochier-Armanet et al., 2008; de la Torre et al., 2008; Spang et al., 2010). Archaeal cultivation studies showed that *Euryarchaeota* with the exception of *Thermoplastmatales*, DHVE-2 cluster, *Thermoccus aggregans*, *Methanopyrus kandleri*, and methanotrophic ANME-1, predominantly produce GDGT-0 (Schouten et al., 2013). GDGTs from samples in Group 2.1 exhibited a dominance of GDGT-0 (**Figure 3**), implicating that *Archaea* in this subgroup may be dominated by *Euryarchaeota*. The presence of GDGTs with cyclopentyl moieties other than GDGT-0 (**Figure 3**) indicated that other *Archaea* may be present as a minor component of the archaeal community in these hot springs.

Thaumarchaeol, originally called crenarchaeol, was considered a biomarker for mesophilic *Crenarchaeota* (Schouten et al., 2002, 2003; Sinninghe Damsté et al., 2002); however, the lineage linked with this lipid has recently been reclassified as a separate phylum of *Archaea* called *Thaumarchaeota* (Brochier-Armanet et al., 2008; Pester et al., 2011; Sinninghe Damsté et al., 2012). Subsequent studies revealed that thaumarchaeol could also be synthesized in geothermal environments by thermophilic *Archaea* (Pearson et al., 2004, 2008; Zhang et al., 2006; Schouten et al., 2007; He et al., 2012; Boyd et al., 2013; Li et al., 2013). Pearson et al. (2004) proposed that thaumarchaeol could relate to some metabolic function of *Archaea*. Indeed, thaumarchaeol was shown to correlate with the abundance of *amoA* gene in soil (Leininger et al., 2006), lake (Buckles et al., 2013), and hot spring (Pitcher et al., 2009a) environments, and also with ammonium, nitrite and/or nitrate concentrations in hot springs (Boyd et al., 2013; Li et al., 2013). Thus thaumarchaeol was proposed as a biomarker for *Thaumarchaeota* involved in chemolithotrophic ammonia oxidation (de la Torre et al., 2008). Previous studies showed that *amoA* genes were present in Yunnan hot springs (Zhang et al., 2008; Jiang et al., 2010; Hedlund et al., 2012) where thaumarchaeol occurred. GDGTs from the hot springs falling in Group 2.2 were dominated by thaumarchaeol, which indicates that *Thaumarchaeota* and the process of ammonia oxidation might occurred in hot springs belonging to this group (**Figure 3**).

Thaumarchaeol abundance showed no correlation with the concentration of ammonium in Yunnan hot springs, this may be due to the contamination of hot spring sediments with crenarchaeol from surrounding soils adjacent to the springs. Also, ammonium concentration was low (below detection limit) in half of the springs studied (**Table 1**) and the small size and uneven distribution of ammonium concentrations may have obscured the correlation between ammonium and thaumarchaeol. In addition, ammonia-oxidizing *Archaea* remove ammonia from their environment and the concentration of ammonia in a spring is dependent on the flux of ammonia, including production/delivery and removal, so it is not surprising that no clear correlation exists.

### FACTORS CONTROLLING GDGT-BASED PROXIES: INSIGHTS ON THE APPLICATION OF PROXIES

In chemically relatively stable environments like the ocean, GDGT-based proxies have been developed to discern temperature or other factors that control archaeal lipid distribution. However, in terrestrial geothermal springs, dynamic geobiological interactions may result in compounded physical, chemical, and biological influences on GDGT-based proxies.

Recent molecular studies have showed that hot springs in Yunnan harbored diverse archaeal populations (Song et al., 2010; Hedlund et al., 2012; Hou et al., 2013) and that the community structures were influenced by the combination of temperature and pH (Hou et al., 2013). In this study, Thaum/(Thaum + GDGT-0) correlated negatively with temperature in Group 1 (**Figure 5A**) and negatively with pH in Group 2 (**Figure 5C**; pH 6–8), suggesting that the relative abundance of *Thaumarchaeota* to *Euryarchaeota* was predominantly affected by temperature in Group 1 and by pH in Group 2. The observation in Group 1 is consistent with that of the Great Basin (Zhang et al., 2006), which may indicate a similar archaeal community structure between Group 1 of Yunnan hot springs and hot springs of the Great Basin reported in Zhang et al. (2006).

In hydrate-impacted marine sediments, the process of anaerobic methane oxidation can be identified by high MI values (MI > 0.85; Zhang et al., 2011). In this study, MI values of most samples in Group 1 (**Figure 5B**) surpassed those of hydrate-impacted marine sediments (Zhang et al., 2011). It is unknown, however, whether anaerobic methane oxidation occurred in Yunnan hot springs because of the lack of data on genes associated with methane-oxidizing organisms (e.g., Orphan et al., 2002) or on carbon isotopes of archaeal membrane lipids (e.g., Hinrichs et al., 1999; Zhang et al., 2003, 2011). On the other hand, MI increased with increasing temperature (**Figure 4B**), which indicates that MI mainly reflects temperature control on GDGT distribution rather than the process of anaerobic methane oxidation in the hot spring environment.

Ring index was found to correlate with temperature or nitrite in subgroups of GDGTs in Tibetan hot springs (Li et al., 2013). In this study, RI did not show any correlation with temperature or other chemical variables in subgroups. In general, GDGTs in Group 2 were dominated by GDGT-0 or thaumarchaeol (two compounds that are not counted in the RI calculation), which resulted in RI values in Group 2 being lower than those in Group 1.

In addition, weak correlations falling below their confidence threshold were observed between GDGT-based proxies and nutrient concentrations (data not shown), which is in contrast to the results from Boyd et al. (2013) and Li et al. (2013). This is possibly due to the small amount of data used in this study compared to those reported by Boyd et al. (2013) and Li et al. (2013).

In summary, samples with different patterns of GDGT distribution were clustered into two major groups (Group 1 and Group 2). MI and Thaum/(Thaum + GDGT-0) were found to correlate with temperature in Group 1, and only Thaum/(Thaum + GDGT-0) correlated with pH in Group 2. The inconsistency of the relationships of organic proxies with environmental parameters using different samples reminds us to be specific when addressing environmental impacts on lipid distributions. This study also demonstrates that the microbial community structure should be taken into consideration in the application of organic proxies.

## Conflict of Interest Statement

The authors declare that the research was conducted in the absence of any commercial or financial relationships that could be construed as a potential conflict of interest.
